# Simultaneous Quantitative Determination of Polyphenolic Compounds in *Blumea balsamifera* (Ai-Na-Xiang, Sembung) by High-Performance Liquid Chromatography with Photodiode Array Detector

**DOI:** 10.1155/2020/9731327

**Published:** 2020-03-18

**Authors:** Daopeng Tan, Zhou Yang, Qianru Zhang, Hua Ling, Yimei Du, Yanliu Lu, Tianpei Xie, Xumei Zhou, Lin Qin, Yuqi He

**Affiliations:** ^1^Key Laboratory of Basic Pharmacalogy of Ministry of Education and Joint International Research Laboratory of Ethnomedicine of Ministry of Education, School of Pharmacy, Zunyi Medical University, Guizhou 563009, China; ^2^State Key Laboratory of Functions and Applications of Medicinal Plants, Guizhou Medical University, Guiyang 550014, China; ^3^Shanghai Nature-Standard Technical Service Co., Ltd., Shanghai 201203, China; ^4^School of Pharmacy, Georgia Campus—Philadelphia College of Osteopathic Medicine, 625 Old Peachtree Rd NW, Suwanee, GA 30024, USA

## Abstract

A high-performance liquid chromatography method was developed for simultaneous quantification of 18 polyphenolic compounds from the leaves of *Blumea balsamifera*, including 17 flavonoids and 1 phenylethanone. The *B. balsamifera* extraction was separated by a Kromasil C_18_ column (250 × 4.6 mm, 5 *μ*m) with a binary gradient mobile phase consisting of acetonitrile and 0.2% aqueous acetic acid. A photodiode array detector (PDA) was used to record the signals of investigated constituents. The linearity, sensitivity, stability, precision, and accuracy of the established assay methods were assessed to meet the requirements of quantitative determination. Samples extracted by reflux in 25 mL of 80% methanol for 30 minutes were selected for the extraction method. The 18 compounds were accurately identified by comparing with the reference compounds. The purity of each peak was confirmed by the base peak in the mass spectrum. The contents of 18 compounds in Blumea samples from four different regions were successfully determined. The results also showed that 3,3′,5,7-tetrahydroxy-4′-methoxyflavanone was the most abundant constituent, which could be used as a potential chemical marker for quality control of *B. balsamifera* and Chinese patent medications containing *B. balsamifera* herb.

## 1. Introduction

*Blumea balsamifera* (Compositae) is a medicinal plant widely growing in China and Southeast Asian countries such as Malaysia, Philippines, Vietnam, and Thailand. In China, it is called as Ai-Na-Xiang, whereas in Philippines it is called Sembung. *B. balsamifera* showed lots of biological effects on stomach, respiratory system [1], nervous system [2], and also showed sudorific [3], antifungal [4], anticancer [5, 6], and antiobesity [4] effects.

Leaves of *B. balsamifera* are sometimes used as tea and cigarettes. It contains abundant flavonoids [7–12] that are a ubiquitous group of polyphenolic substances. Similar to the whole plant of *B. balsamifera*, flavonoids from leaves of *B. balsamifera* also showed broad pharmacological activities such as radical scavenging [10, 13], anticancer [14, 15], plasmin-inhibition [16], liver-protection [17] and xanthine oxidase inhibitory effects [12].

Quality control is crucial to guarantee the safety and efficacy of the utilization of herbal medicines. Unlike the synthetic drugs, the effectiveness of herbal medicines may be attributed to the overall effect of several components rather than a single component. Moreover, the synergistic effect between components is also related to herb efficacy. Thus, the quality evaluation of herbal medicine is very difficult and requires the information of bioactive components as much as possible. Several studies have reported the determination of constituents in *B. balsamifera* [18]. However, most of them have focused on only a few components. To the best of our knowledge, only one article showed a determination of 5 flavonoids in the leaves of *B. balsamifera* [19]; however, it requires a tedious sample preparation procedure.

High-performance liquid chromatography (HPLC) coupled with various detectors such as ultraviolet-visible (UV) detection [20], photodiode array (PDA) detection [21], and mass spectrometry (MS) [22] are accepted methods applied in herb quality control. Among them, HPLC coupled with PDA (HPLC-PDA) is the preferred method used in pharmaceutical industry because of its high efficiency and low cost.

In the present study, we developed a simple HPLC-PDA method to simultaneously determine 18 bioactive compounds in the leaves of *B. balsamifera*. The established method was simple, reliable, and high throughout and would be potentially used to control the quality of *B. balsamifera* precisely.

## 2. Materials and Methods

### 2.1. Materials and Chemicals

Four batches of the leaves of *B. balsamifera* were collected from Guizhou, Guangxi, Yunnan province of China. The sample materials were identified by Dr. Daopeng Tan as the leaves of *B. balsamifera*.

**Figure 1:**
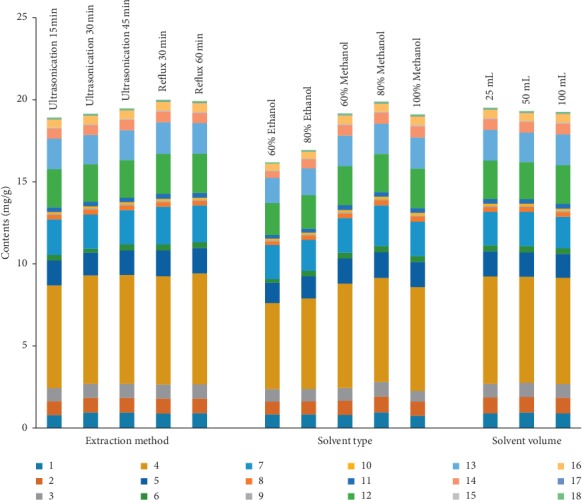
Effects of the extraction method, solvent type, and solvent volume on the extraction efficiency of 18 constituents in the leaves of *B. balsamifera* (**1**, Rutin; **2**, Hyperoside; **3**, Isoquercitrin; **4**, 3,3′,5,7-Tetrahydroxy-4′-methoxyflavanone; **5**, 3′,5,5′,7-Tetrahydroxyflavanone; **6**, Quercetin; **7**, 3,3′,4′,5-Tetrahydroxy-7-methoxyflavanone; **8**, Chrysosplenol C; **9**, Diosmetin; **10**, Tamarixetin; **11**, 3,5,7-Trihydroxy-3′,4′-dimethoxyflavone; **12**, 3,3′,5-Trihydroxy-4′,7-dimethoxyflavanone; **13**, Blumeatin; **14**, Rhamnetin; **15**, 3′,4′,5-Trihydroxy-3,7-dimethoxyflavone; **16**, Xanthoxylin; **17**, Ombuin; **18**, 3,5-Dihydroxy-3′,4′,7-trimethoxyflavone). When one parameter was determined, the others were set at the default value.

A total of 18 reference compounds including 17 flavonoids and 1 phenylethanone (Figure 1) were isolated previously from the leaves of *B. balsamifera* in our laboratory and elucidated by NMR and MS [23, 24]. The purities of these reference standards were higher than 98.0% checked by the HPLC method.

HPLC grade acetonitrile (Merck, Germany) was used as the mobile phase. All other reagents were at least analytical grade (Jinhuada Chemical Factory, Guangzhou, China). Water was purified using a Milli-Q water purification system (Millipore, USA).

### 2.2. Sample Preparation

The ground dried leaves of *B. balsamifera* were dried and pulverized into a homogeneous powder mixture (60 meshes). The powder (0.6 g) was extracted by reflux in 80% methanol (25 mL) for 30 min. The extracted sample solution was filtered with a 0.45 *μ*m membrane prior to the HPLC analysis.

The reference standards were accurately weighed and dissolved in methanol to prepare mixed standard stock solution. Consisted of each reference compounds **1**–**18** in the mixed standard stock solution were 233.2 *μ*g/mL, 211.1 *μ*g/mL, 195.3 *μ*g/mL, 1669.0 *μ*g/mL, 330.5 *μ*g/mL, 57.3 *μ*g/mL, 443.5 *μ*g/mL, 51.6 *μ*g/mL, 25.8 *μ*g/mL, 28.1 *μ*g/mL, 68.3 *μ*g/mL, 534.2 *μ*g/mL, 447.9 *μ*g/mL, 125.5 *μ*g/mL, 15.7 *μ*g/mL, 123.8 *μ*g/mL, 16.9 *μ*g/mL, and 20.2 *μ*g/mL, respectively. The mixed standard stock solution was stored at 4°C without exposure of light for further analysis. Working standard solutions used to prepare calibration curves and check recovery were prepared by diluting the standard stock solution into serial concentrations with methanol. The standard solutions were filtered through a 0.45 *μ*m membrane prior to the HPLC analysis.

### 2.3. Chromatography Parameters

The HPLC system consists of a Waters 2995 controller and 2998 Photodiode Array detector. The separation was performed on an Elite Kromasil C_18_ column (250 mm × 4.6 mm, 5 *μ*m). The flow rate was set at 1.0 mL/min, with the column temperature set at 25°C, and detective wavelength was set at 254 and 289 nm. The acetonitrile and water containing 0.2% acetic acid were employed as mobile phases A and B, respectively. The binary gradient program was set as follows: 0–10 min, 80% of B; 10–15 min, 80 to 75% of B; 15–25 min, 75% of B; 25–50 min, 75 to 35% of B; and equilibrated for 10 min before the next injection. The injection volume was 10 *μ*L. Data were collected and visualized by Waters Empower Chemstation Software.

The chromatographic peak purity analysis was performed on an Agilent 1290 UPLC system coupled with a SCIEX Triple TOF 4600 mass spectrometer equipped with an ESI interface. The optimized MS conditions were as follows: TOF mass range between 50 and 1700, curtain gas 35 psig, ion spray voltage floating −4500/5000 kV, and ion source temperature 500°C. The collision energy was set at 10 V to obtain more fragment information.

### 2.4. Method Validation

The linearity, limit of detection (LOD), limit of quantification (LOQ), precision, repeatability, stability, and recovery were checked for method validation.

## 3. Results and Discussion

### 3.1. Optimization of the Extraction Method

Four factors (including extraction methods, extraction solvents, solvent volume, and extraction time) were evaluated to get the most efficient extraction protocol. Sonication (15, 30, and 45 min) and reflux (30 and 60 min) in factorial experiments using 80% methanol and the reflux for 30 min showed the best extraction ability. Moreover, among 60% methanol, 80% methanol, 100% methanol, 60% ethanol, and 80% ethanol, 80% methanol was the best solvent mixture and produced more chromatographic peaks. Furthermore, 25 mL of 80% methanol showed the best extraction efficiency compared to solvent volumes 50 and 100 mL. Finally, samples extracted by reflux in 25 mL of 80% methanol for 30 min were selected for the extraction method (Figure 1).

### 3.2. Optimization of HPLC Parameters

**Figure 2:**
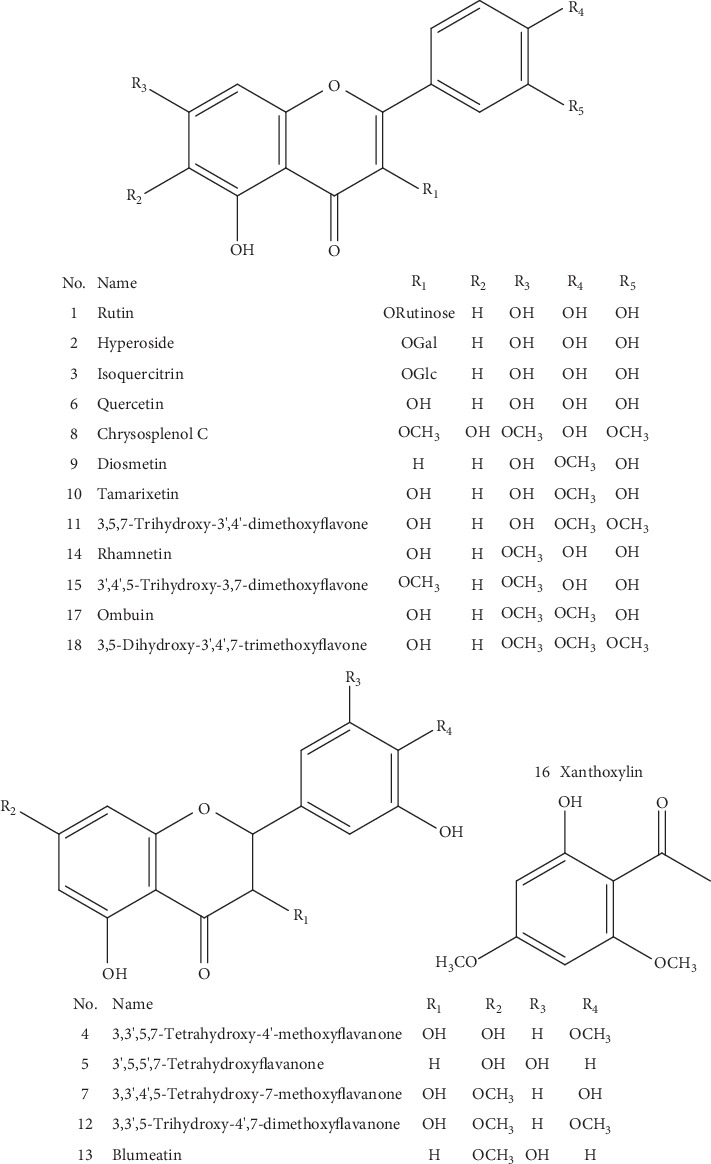
The structures of 18 reference standards isolated from the leaves of *B. balsamifera*.

**Figure 3:**
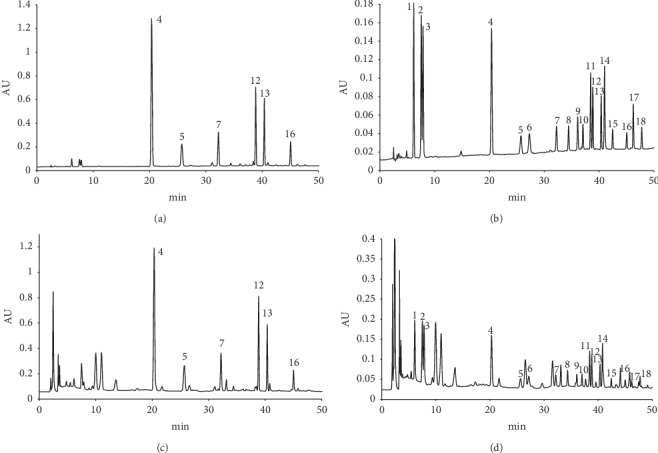
HPLC chromatographs. ((a) Reference standards at 289 nm; (b) reference standards at 254 nm; (c) the sample of batch no. GZ20151001 at 289 nm; (d) the sample of batch no. GZ20151001 at 254 nm; **1**, Rutin; **2**, Hyperoside; **3**, Isoquercitrin; **4**, 3,3′,5,7-Tetrahydroxy-4′-methoxyflavanone; **5**, 3′,5,5′,7-Tetrahydroxyflavanone; **6**, Quercetin; **7**, 3,3′,4′,5-Tetrahydroxy-7-methoxyflavanone; **8**, Chrysosplenol C; **9**, Diosmetin; **10**, Tamarixetin; **11**, 3,5,7-Trihydroxy-3′,4′-dimethoxyflavone; **12**, 3,3′,5-Trihydroxy-4′,7-dimethoxyflavanone; **13**, Blumeatin; **14**, Rhamnetin; **15**, 3′,4′,5-Trihydroxy-3,7-dimethoxyflavone; **16**, Xanthoxylin; **17**, Ombuin; **18**, 3,5-Dihydroxy-3′,4′,7-trimethoxyflavone).

**Figure 4:**
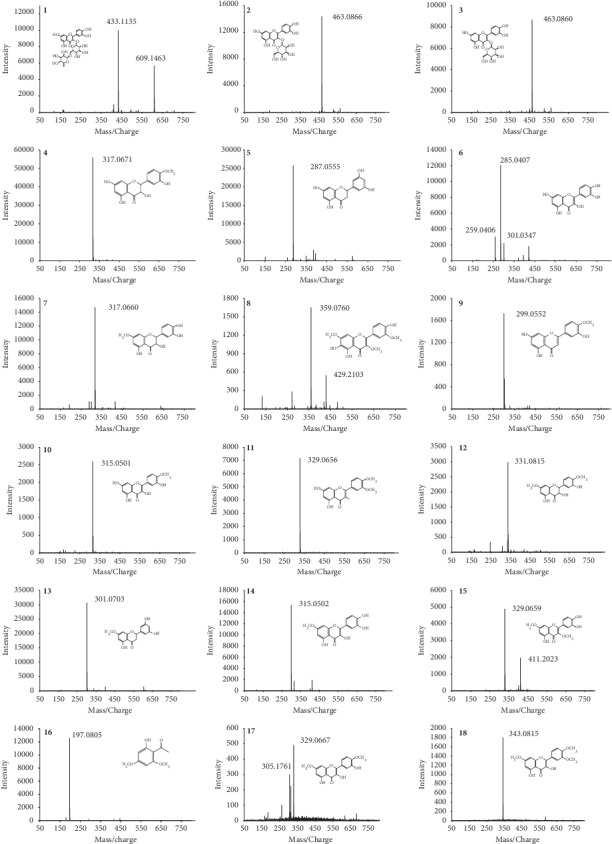
MS spectrum (ESI^−^ for **1**, Rutin; **2**, Hyperoside; **3**, Isoquercitrin; **4**, 3,3′,5,7-Tetrahydroxy-4′-methoxyflavanone; **5**, 3′,5,5′,7-Tetrahydroxyflavanone; **6**, Quercetin; **7**, 3,3′,4′,5-Tetrahydroxy-7-methoxyflavanone; **8**, Chrysosplenol C; **9**, Diosmetin; **10**, Tamarixetin; **11**, 3,5,7-Trihydroxy-3′,4′-dimethoxyflavone; **12**, 3,3′,5-Trihydroxy-4′,7-dimethoxyflavanone; **13**, Blumeatin; **14**, Rhamnetin; **15**, 3′,4′,5-Trihydroxy-3,7-dimethoxyflavone; **17**, Ombuin; **18**, 3,5-Dihydroxy-3′,4′,7-trimethoxyflavone; ESI^+^ for **16**, Xanthoxylin).

To get an accepted resolution, separation parameters including analytical column, mobile phases, and elution gradient were assessed. The Elite Kromasil C_18_ column (250 mm × 4.6 mm, 5 *μ*m) got the best resolution among all investigated columns. By comparing the strength of the response signals, the wavelength of 254 nm was selected for compounds **1**–**3**, **6**, **8**–**11**, **14**, **15**, **17**, and **18**, while 289 nm was selected for compounds **4**, **5**, **7**, **12**, **13**, and **16** shown in Figure 2. In order to simultaneously detect all the 18 aforementioned compounds, in the analysis, both 254 nm and 289 nm were used concurrently. The typical HPLC chromatograms of the mixed standard solution are shown in Figure 3. The chromatographic purities of the 18 polyphenolic compound peaks were determined at multiple wavelengths by HPLC-PDA spectroscopy and the HPLC-MS method. In the HPLC-PDA spectroscopy, the purity factor of all peaks is within the calculated threshold limit. In the total ion chromatography obtained from HPLC-MS, we extracted the MS spectrum of the 18 peaks, and the *m*/z values of base peaks corresponded with their molecular weight information (Figure 4).

### 3.3. Validation of the Developed Method

The established method was validated in terms of linearity, precision, stability, and accuracy. Linear regression equations (e.g., *y* = *ax* + *b*) were constructed by plotting peak areas (*y*) of each analyte against analyte concentrations (*x*; *μ*g/mL). The LOD and LOQ values were determined based on S/N of 3 and 10, respectively.  Table 1 summarizes the linearity, test range, limit of detection (LOD), and limit of quantification (LOQ). The linearity is indicated by the correlation coefficient (*R*^2^). All analytes showed linearity with *R*^2^ between 0.9996 and 1.0000 in the test range. The LODs and LOQs for the 18 tested reference standards were around 0.02–1.32 *μ*g/mL and 0.05–5.29 *μ*g/mL, respectively.

Precision was evaluated by using intraday and interday variability. The intraday variability was assessed at three different concentration levels (low, medium, and high) with six replicates at each level within one day. The interday variability was tested in triplicate on the consecutive three days. The variations (RSD%) for intra- and interday precision are shown in Table 2. The overall intra- and interday variations were less than 3.33%. The repeatability was conducted using six replicates of the same sample, and the variations of repeatability were less than 2.98%. The stability of the sample solution was investigated at 0, 2, 4, 8, 12, and 18 h. The RSD% of peak areas of the analyzed compounds in the actual samples was ≤3.02%, indicating that the analyzed compounds in the samples were stable at least 18 h.

The recovery test was used to evaluate the accuracy of the method. Three different concentration levels (80%, 100%, and 120% of the concentration of the targets in a random sample) of the standard solutions were added into an actual sample. Triplicates were done for each level (0.33 g, 0.30 g, and 0.27 g *B. balsamifera* powder). The recovery of each spiked reference standards was calculated by the formula recovery% = [(found amount−original amount)/spiked amount] × 100%. The spike recoveries for 18 analyzed samples were 95.03–109.92% (Table 3).

Above data demonstrated that the developed HPLC-PDA method was precise and accurate for quantitative determination of the 18 tested constituents in *B. balsamifera.*

### 3.4. Sample Determination

Herbs contain multiple bioactive components. Controlling the quality of herbs using a unique constituent is not an accepted method; thus, a tailored analytical method for each herb to evaluate multiple bioactive components simultaneously is needed. In the current study, we developed a simple and accurate assay method for simultaneous determination of 18 major compounds in the leaves of *B. balsamifera.*

The validated HPLC-PDA method was applied to the simultaneous determination of 18 bioactive components in the leaves of *B. balsamifera* collected from different regions in China. The results are shown in Table 4.

The content of compound **4** (3,3′,5,7-tetrahydroxy-4′-methoxyflavanone) showed the highest content among all tested compounds. Furthermore, the content of each studied components showed marked variations among different regions (Table 4). Therefore, it is necessary to establish a good agricultural practice (GAP) standard to grow medicinal plants with stable and consistent chemical ingredients.

## 4. Conclusion

In the present study, a simple and accurate HPLC-PDA analytic method was developed to quantify 18 bioactive compounds, simultaneously, in the leaves of *B. balsamifera*. This established HPLC method is helpful to improve the quality control of the leaves of *B. balsamifera* and related downstream products.

## Figures and Tables

**Table 1:** Linearity and sensitivity of the HPLC analysis.

No.	Compound name	Calibration curve^a^	*R* ^2^	Linear range (*μ*g/mL)	LOD^b^ (*μ*g/mL)	LOQ^c^ (*μ*g/mL)
1	Rutin	*y* = 36771*x* + 4732.7	0.9997	0.37∼139.92	0.18	0.37
2	Hyperoside	*y* = 45866*x* − 25252	0.9999	0.34∼126.65	0.10	0.34
3	Isoquercitrin	*y* = 45714*x* − 5732.6	0.9999	0.31∼117.20	0.10	0.31
4	3,3′,5,7-Tetrahydroxy-4′-methoxyflavanone	*y* = 6982.1*x* − 19647	0.9999	2.67∼1001.40	0.89	2.67
5	3′,5,5′,7-Tetrahydroxyflavanone	*y* = 7495.0*x* − 9796.1	1.0000	6.61∼330.47	1.32	5.29
6	Quercetin	*y* = 50230*x* − 22534	0.9999	1.15∼57.34	0.23	0.92
7	3,3′,4′,5-Tetrahydroxy-7-methoxyflavanone	*y* = 5499.5*x* − 15331	0.9997	3.55∼443.52	0.88	3.55
8	Chrysosplenol C	*y* = 39377*x* − 14116	0.9998	0.21∼51.55	0.07	0.21
9	Diosmetin	*y* = 1.0 *E* + 5*x* − 14906	1.0000	0.10∼36.06	0.03	0.10
10	Tamarixetin	*y* = 64551*x* − 12598	0.9997	0.11∼28.06	0.04	0.11
11	3,5,7-Trihydroxy-3′,4′-dimethoxyflavone	*y* = 70721*x* − 19615	0.9999	0.11∼95.68	0.04	0.11
12	3,3′,5-Trihydroxy-4′,7-dimethoxyflavanone	*y* = 7271.0*x* − 5822.3	1.0000	0.86∼747.94	0.29	0.86
13	Blumeatin	*y* = 7339.9*x* − 8361.5	1.0000	0.72∼627.09	0.24	0.72
14	Rhamnetin	*y* = 46787*x* − 11774	1.0000	0.20∼175.73	0.07	0.20
15	3′,4′,5-Trihydroxy-3,7-dimethoxyflavone	*y* = 88162*x* − 11165	0.9996	0.06∼22.04	0.02	0.06
16	Xanthoxylin	*y* = 10671*x* − 5373.4	1.0000	0.99∼173.27	0.33	0.99
17	Ombuin	*y* = 1.9 *E* + 5*x* − 18006	0.9999	0.07∼23.66	0.02	0.05
18	3,5-Dihydroxy-3′,4′,7-trimethoxyflavone	*y* = 74640*x* − 9102.7	0.9999	0.08∼20.18	0.03	0.08

*y* is the peak area and *x* is the concentration of compound (*μ*g/mL). ^b^LOD refers to the limit of detection, S/N = 2.3–3.6 : 1. ^c^LOQ refers to the limit of quantification, S/N = 8.1–10.3 : 1.

**Table 2:** The results of precision, repeatability, and stability.

No.	Intraday (RSD, %, *n* = 6)			Interday (*n* = 6)			Repeatability (*n* = 6)	Stability (*n* = 6, 18 h)
	Low	Medium	High	Low	Medium	High	RSD (%)	RSD (%)
1	1.08	1.03	0.77	1.98	2.17	1.89	1.46	2.31
2	0.31	0.40	0.39	1.14	1.07	0.84	1.03	1.26
3	0.52	0.70	0.58	1.42	1.17	0.94	1.01	1.11
4	1.86	1.86	1.60	2.13	2.30	1.92	1.97	2.44
5	0.50	0.68	0.61	0.62	0.62	0.66	0.56	0.79
6	0.57	0.59	0.60	1.04	0.88	0.99	0.69	0.90
7	0.86	0.96	0.73	1.04	0.76	0.86	0.70	0.87
8	1.08	1.38	1.28	1.73	1.74	2.09	1.38	1.27
9	1.05	1.16	1.11	1.68	3.33	2.96	2.84	3.02
10	0.98	1.53	0.98	1.40	1.21	1.28	1.04	1.81
11	0.57	0.67	0.56	0.74	0.74	0.77	0.54	0.70
12	0.65	0.65	0.50	0.79	0.79	0.52	0.60	0.63
13	0.59	0.71	0.56	0.32	0.52	0.32	0.52	0.66
14	0.70	0.66	0.62	0.52	0.87	1.11	0.82	0.93
15	2.39	2.19	1.90	0.93	1.12	1.40	1.25	1.78
16	1.21	1.13	1.02	0.64	0.58	0.68	1.31	1.58
17	2.35	3.33	3.06	1.62	1.70	1.47	2.98	1.70
18	0.99	0.60	0.45	0.67	0.70	0.86	0.65	0.74

**Table 3:** Recovery of the targets (*n* = 3).

No.	Original (mg)	Spiked (mg)	Found (mg)	Recovery (%)^a^	RSD (%)^b^
1	0.36	0.23	0.59	100.95 ± 3.95	3.91
	0.30	0.29	0.61	104.00 ± 2.67	2.56
	0.24	0.35	0.59	102.15 ± 3.37	3.30

2	0.33	0.21	0.55	102.84 ± 4.08	3.97
	0.28	0.26	0.56	104.51 ± 2.94	2.81
	0.22	0.32	0.54	102.64 ± 2.41	2.34

3	0.32	0.20	0.52	103.76 ± 3.67	3.54
	0.27	0.24	0.53	105.68 ± 3.02	2.86
	0.21	0.29	0.51	103.49 ± 2.38	2.30

4	2.40	1.67	4.02	96.90 ± 4.18	4.31
	2.04	2.09	4.12	99.95 ± 2.63	2.63
	1.60	2.50	4.09	99.53 ± 2.31	2.32

5	0.54	0.33	0.88	100.70 ± 4.80	4.76
	0.46	0.41	0.89	103.53 ± 3.67	3.55
	0.36	0.50	0.87	102.48 ± 3.64	3.56

6	0.11	0.06	0.17	103.95 ± 4.36	4.19
	0.10	0.07	0.17	106.42 ± 0.32	0.30
	0.08	0.09	0.17	109.92 ± 0.51	0.46

7	0.74	0.44	1.21	104.71 ± 1.64	1.57
	0.63	0.55	1.17	96.69 ± 3.77	3.89
	0.50	0.67	1.13	95.37 ± 2.22	2.32

8	0.10	0.05	0.16	108.99 ± 3.63	3.33
	0.09	0.06	0.16	108.76 ± 0.88	0.81
	0.07	0.08	0.15	107.31 ± 1.03	0.96

9	0.03	0.03	0.05	103.56 ± 2.84	2.74
	0.02	0.03	0.06	109.35 ± 0.45	0.41
	0.02	0.04	0.06	104.19 ± 0.37	0.36

10	0.05	0.03	0.08	101.56 ± 3.61	3.56
	0.04	0.04	0.08	108.86 ± 1.50	1.38
	0.03	0.04	0.08	107.88 ± 0.30	0.27

11	0.10	0.07	0.17	102.90 ± 4.55	4.42
	0.09	0.09	0.18	104.61 ± 3.51	3.36
	0.07	0.10	0.18	103.53 ± 2.47	2.39

12	0.84	0.53	1.39	102.48 ± 4.30	4.20
	0.72	0.67	1.41	104.43 ± 3.43	3.28
	0.56	0.80	1.39	103.05 ± 2.48	2.41

13	0.67	0.45	1.13	102.81 ± 4.19	4.08
	0.57	0.56	1.16	104.67 ± 3.05	2.91
	0.45	0.67	1.14	103.34 ± 2.43	2.36

14	0.24	0.13	0.36	97.59 ± 3.55	3.64
	0.20	0.16	0.36	102.75 ± 1.42	1.39
	0.16	0.19	0.36	106.26 ± 0.52	0.49

15	0.02	0.02	0.04	103.40 ± 4.19	4.05
	0.02	0.02	0.04	104.65 ± 3.03	2.90
	0.01	0.02	0.04	100.95 ± 2.78	2.76

16	0.19	0.12	0.31	95.85 ± 2.17	2.26
	0.16	0.16	0.31	95.64 ± 2.64	2.76
	0.13	0.19	0.31	95.03 ± 2.21	2.33

17	0.01	0.02	0.03	105.40 ± 0.99	0.93
	0.01	0.02	0.03	107.31 ± 0.15	0.14
	0.01	0.03	0.03	109.30 ± 0.52	0.47

18	0.03	0.02	0.05	104.16 ± 4.70	4.52
	0.03	0.03	0.05	105.74 ± 3.09	2.93
	0.02	0.03	0.05	105.15 ± 2.54	2.42

^a^Recovery% = [(found amount−original amount)/spiked amount] × 100%. ^b^RSD% = (SD/mean) × 100%.

**Table 4:** The contents of the 18 targets in the leaves of *B. Balsamifera* (mg/g, mean ± SD, *n* = 3).

No.	GZ20151001	GZ20151002	GX20151001	YN20151001
1	0.97 ± 0.001	1.01 ± 0.002	1.00 ± 0.000	0.47 ± 0.012
2	0.91 ± 0.003	0.82 ± 0.002	0.45 ± 0.007	0.47 ± 0.006
3	0.87 ± 0.003	0.77 ± 0.003	0.43 ± 0.006	0.45 ± 0.002
4	6.54 ± 0.050	12.61 ± 0.193	4.39 ± 0.046	3.81 ± 0.106
5	1.51 ± 0.007	0.96 ± 0.006	0.96 ± 0.012	2.38 ± 0.003
6	0.32 ± 0.003	0.32 ± 0.000	0.28 ± 0.002	0.33 ± 0.002
7	2.07 ± 0.027	2.48 ± 0.002	1.42 ± 0.010	3.29 ± 0.001
8	0.27 ± 0.004	0.34 ± 0.002	0.28 ± 0.001	0.17 ± 0.001
9	0.07 ± 0.001	0.08 ± 0.000	0.06 ± 0.000	0.01 ± 0.000
10	0.13 ± 0.001	0.13 ± 0.001	0.07 ± 0.001	0.11 ± 0.000
11	0.29 ± 0.003	0.20 ± 0.001	0.23 ± 0.001	0.28 ± 0.000
12	2.34 ± 0.020	2.36 ± 0.004	1.78 ± 0.012	1.80 ± 0.001
13	1.87 ± 0.012	2.17 ± 0.004	1.59 ± 0.009	1.31 ± 0.000
14	0.66 ± 0.005	0.48 ± 0.001	0.30 ± 0.003	0.31 ± 0.000
15	0.06 ± 0.001	0.06 ± 0.000	0.03 ± 0.000	0.06 ± 0.000
16	0.53 ± 0.001	1.14 ± 0.003	0.50 ± 0.003	0.35 ± 0.000
17	0.03 ± 0.001	0.02 ± 0.000	0.03 ± 0.000	0.01 ± 0.000
18	0.09 ± 0.001	0.10 ± 0.002	0.00 ± 0.000	0.06 ± 0.000

## Data Availability

The research data generated from this study are included within the article.

## Abbreviations

HPLC: High-performance liquid chromatography
PDA: Photodiode array detector
*B. balsamifera*: *Blumea balsamifera*
LOD: Limit of detection
LOQ: Limit of quantification
RSD: Relative standard deviation.
